# Soundscape enrichment increases larval settlement rates for the brooding coral *Porites astreoides*

**DOI:** 10.1098/rsos.231514

**Published:** 2024-03-13

**Authors:** Nadège Aoki, Benjamin Weiss, Youenn Jézéquel, Weifeng Gordon Zhang, Amy Apprill, T. Aran Mooney

**Affiliations:** ^1^ Department of Biology, Woods Hole Oceanographic Institution, 266 Woods Hole Road, Falmouth, MA 02543, USA; ^2^ Department of Earth, Atmospheric, and Planetary Sciences, Massachusetts Institute of Technology, 77 Massachusetts Avenue, Cambridge, MA 02139, USA; ^3^ Department of Applied Ocean Physics and Engineering, Woods Hole Oceanographic Institution, 266 Woods Hole Road, Woods Hole, Falmouth, MA 02543, USA; ^4^ Department of Marine Chemistry and Geochemistry, Woods Hole Oceanographic Institution, 266 Woods Hole Road, Woods Hole, Falmouth, MA 02543, USA

**Keywords:** acoustic playback, coral reefs, recruitment, restoration, marine invertebrates, hearing

## Abstract

Coral reefs, hubs of global biodiversity, are among the world’s most imperilled habitats. Healthy coral reefs are characterized by distinctive soundscapes; these environments are rich with sounds produced by fishes and marine invertebrates. Emerging evidence suggests these sounds can be used as orientation and settlement cues for larvae of reef animals. On degraded reefs, these cues may be reduced or absent, impeding the success of larval settlement, which is an essential process for the maintenance and replenishment of reef populations. Here, in a field-based study, we evaluated the effects of enriching the soundscape of a degraded coral reef to increase coral settlement rates. *Porites astreoides* larvae were exposed to reef sounds using a custom solar-powered acoustic playback system. *Porites astreoides* settled at significantly higher rates at the acoustically enriched sites, averaging 1.7 times (up to maximum of seven times) more settlement compared with control reef sites without acoustic enrichment. Settlement rates decreased with distance from the speaker but remained higher than control levels at least 30 m from the sound source. These results reveal that acoustic enrichment can facilitate coral larval settlement at reasonable distances, offering a promising new method for scientists, managers and restoration practitioners to rebuild coral reefs.

## 1. Introduction

Coral reef ecosystems are centres of marine biodiversity, providing ecosystem services such as coastline protection, fisheries provisioning and tourism to millions of people around the world [1]. Unfortunately, reefs around the globe have experienced precipitous declines in recent decades owing to anthropogenic and climate change-related stressors [2,3]. These stressors have led to extensive biodiversity losses on reefs and accelerated erosion of the three-dimensional coral structures that provide habitat structure and substrate for organisms and protect coastal communities from storm hazards [4,5]. Although some reefs have shown capacity to recover from isolated disturbance events [6], the multi-pronged nature and breakneck pace of modern reef degradation will probably outstrip natural rates of ecological recovery for corals over the next century without mediation [7]. Given the urgency of the coral reef crisis, there is a growing understanding and emphasis within the scientific community of the need to support the recovery of foundational hermatypic coral species through human interventions [8].

Like other benthic organisms, corals rely on the processes of larval dispersal and settlement to maintain their existing populations, replenish depleted reefs and provide new sources of genetic variation [9,10]. Coral larvae use a wide range of chemical and biophysical cues from the environment to locate favourable substrates on which to permanently settle and metamorphize from mobile planulae into sessile polyps [11]. These settlement cues may be chemical in nature, produced by crustose coralline algae or microbial biofilms [12,13], or they may be related to other environmental characteristics such as pressure, light, colour, microtopography and sound [14–17]. A concern for declining coral populations is that on degraded reefs, these cues may be absent or below the detection thresholds necessary to elicit larval behavioural responses, leading to reduced settlement [18–21]. In order to support reef rebuilding efforts, there is a crucial need for a more holistic understanding of the cues that drive and attract coral larvae to settle under different environmental conditions [22].

Researchers are increasingly recognizing the ecological importance of soundscapes, or the ambient sound environment, for marine ecosystems [23,24]. Underwater sounds can propagate efficiently over relatively long distances (metres up to kilometres, depending on the source level and propagation conditions such as depth) and have the advantage of being detectable when light and other cues are limited. Coral reefs are acoustically active habitats, with many varied biological and physical sound sources. The communication and ancillary sounds of fishes, snapping shrimp pulses, waves and local weather events all contribute to the natural soundscape [25,26]. These acoustic cues inherently contain information about local community composition and habitat quality. Biological sound cues, particularly low-frequency fish sounds, are known to be indicators of healthy, biodiverse reefs [27,28], while degraded reef soundscapes appear to show lower overall levels of biological sound production and score lower on indices measuring acoustic richness and complexity [20,29,30]. Sound detection is in turn a sensory mode broadly used by adult and larval stages of many marine organisms, including mammals, fishes and various marine invertebrates, to navigate and communicate underwater [31,32]. Given the central sensory importance of soundscapes to coral reefs, consideration has begun to be directed towards the integration of acoustic cues into reef restoration practises [33].

Acoustic enrichment, or the use of underwater speaker systems to play recordings of sounds from healthy coral reefs, is a technique that has been explored to enhance degraded soundscapes and attract settling larvae of various species to restoration sites [34]. Initial studies have successfully used acoustic enrichment to increase the retention of settlement-stage fishes on reefs [35], enhance the settlement of oyster larvae in the laboratory and on reefs [36–39] and accelerate the development in post-larval lobsters [40], underscoring the promise of aquatic acoustic enrichment methods for an array of taxa.

Coral settlement has not previously been targeted by acoustic enrichment experiments, but studies have confirmed that coral larvae are sound-sensitive. Phonotaxis (orientation and movement towards a sound source) was first documented in larvae of the stony coral *Montastrea faveolata* [15]. Subsequently, *Orbicella faveolata* and *Porites astreoides* larvae have been shown to settle preferentially on reef sites with higher levels of ambient low-frequency sound and crepuscular fish chorusing [41,42]. The sensory mechanism by which coral larvae detect sound remains unknown, although it has been suggested that epidermal cilia on the external surface of the larvae may contribute to this mechanosensory function [15]. Taken together, these results suggest that it could be possible to promote coral settlement by reintroducing sound cues to sites that currently lack the acoustic signature of a healthy reef. Given the keystone nature of corals on tropical reefs, there is an opportunity for experiments to examine whether acoustic enrichment can influence coral larval settlement, which will help inform the use of including sound in reef intervention efforts.

Here, we sought to investigate the role of underwater soundscapes in coral settlement and to address the range and practical implications of acoustic enrichment as a reef restoration tool. We developed a custom solar-powered acoustic playback system and used 10 years of reef recordings from the United States Virgin Islands (USVI) [28,43] to expose larvae of the coral *P. astreoides* at a degraded reef site to a soundscape recorded at a historically higher quality, acoustically rich site. Using controlled chambers with filtered seawater to isolate larvae from external chemical cues, we assessed how settlement rates varied when larvae were placed at reef sites with and without an artificially enriched soundscape. We further addressed the ecological role(s) of this enrichment by examining how larval settlement rates varied with respect to the timing and distance of larval exposure to the sound source. We hypothesized that (i) *P. astreoides* larval settlement rates would be higher on both an unenriched high-quality reef and an enriched degraded reef compared with an unenriched degraded reef, (ii) larval settlement at the playback site would decrease as a function of distance from the speaker, and (iii) the impact of acoustic enrichment on settlement would be highest during the first 24 h of exposure to the sound cues.

## 2. Material and methods

### 2.1. Coral collection and larval spawning

Adult colonies (*n* = 30) of the hermaphroditic brooding coral *P. astreoides* were manually collected from the National Park on the south side of St John, USVI at depths ranging between 2 and 12 m from reefs separate from our experimental sites. We selected *P. astreoides* as a study subject based on a previous experiment that documented responses in these larvae to variable soundscapes [42], as well as the widespread local abundance and hardiness of this species to collection and handling. The colonies were collected one week prior to the June 2022 new moon and maintained in shaded outdoor flow through seawater tables at the Virgin Islands Environmental Resource Station. Corals were monitored for the release of planulae in June and July over two respective 6-day periods centred around the new moon [44]. During these periods, individual colonies were placed overnight in 5 μm mesh-lined containers, and the water level was lowered such that colonies remained immersed, but any released larvae were retained by the mesh. Colonies were checked daily, and any swimming larvae were transferred via pipette to clean Petri dishes filled with 0.22 μm filtered seawater. All colonies were returned to the reefs from which they were collected following the conclusion of the experiments.

The experiment was repeated twice, with new larvae collected during the June and July spawning events; a total of 398 larvae were collected in June and 556 larvae were collected in July. All actively swimming planulae from a given month’s spawning period were pooled and divided into groups of 15 (June) or 20 (July) larvae and randomly assigned to one of 24 (June) or 32 (July) total treatment cups for the acoustic experiment.

### 2.2. Experimental chambers

Following the methods outlined by Lillis *et al*. [42], larvae were isolated from extraneous settlement cues during the experiments in 140 ml polypropylene pre-soaked settlement cups. These chambers were filled with 0.22 μm filtered seawater to remove grazers and water-borne microbial cues but remained transparent to sound and light. Each settlement chamber contained a preconditioned (one month in local seawater) clay kiln stilt (3.8 cm diameter), which served as a standardized settlement surface. Dissolved O_2_ levels in the cups were measured prior to the addition of larvae and immediately upon retrieval using an oxygen meter (Orion Star A329; Thermo Scientific) to ensure that available oxygen in the cups remained within the expected ranges of diel variability for coral reefs [45].

### 2.3. Experimental sites

**Figure 1.**
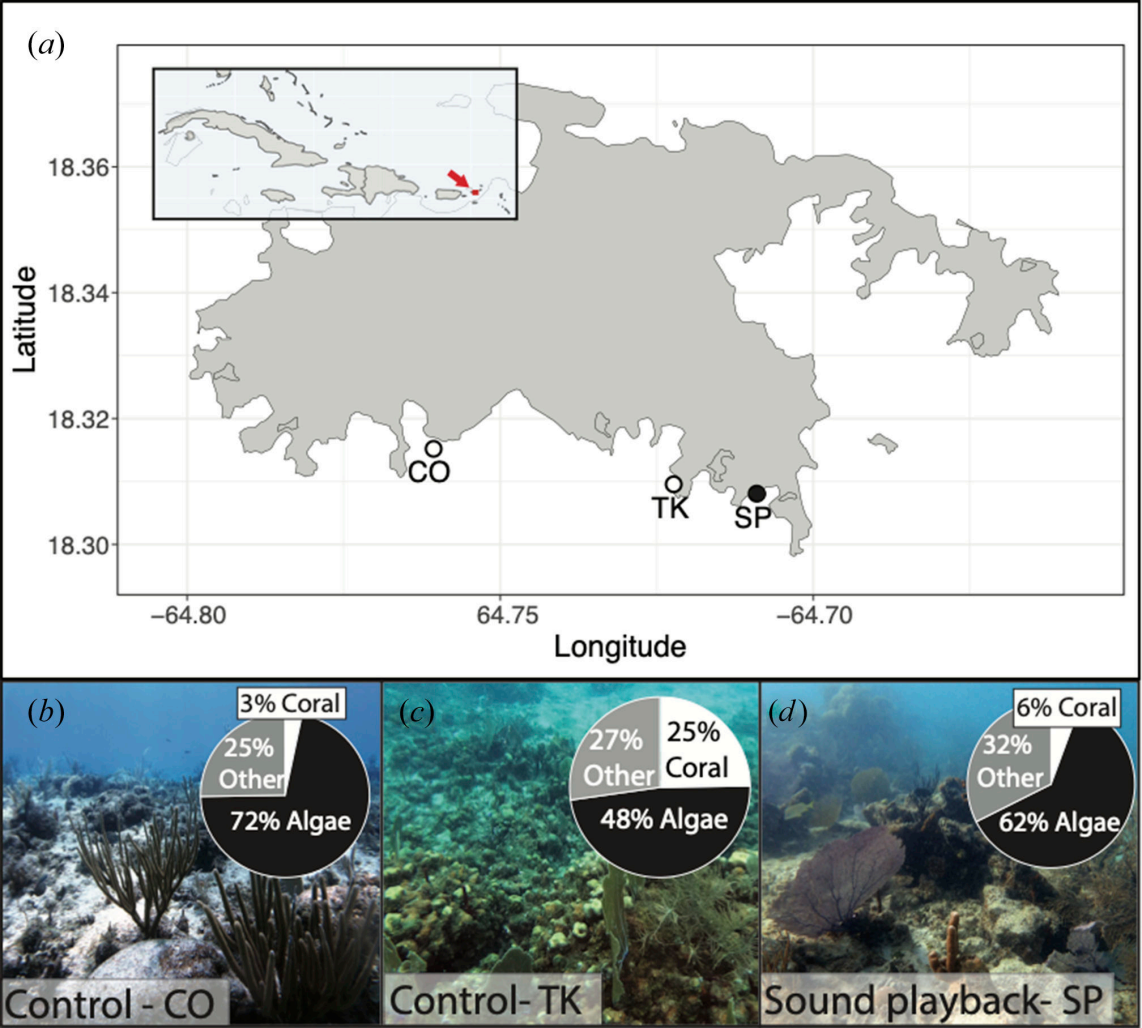
Overview of experimental coral reef sites. (*a*) Map of St John, USVI with experimental sites within the Virgin Islands National Park (Cocoloba, Tektite and Salt Pond) marked. White circles denote control sites and black circle denotes experimental playback site. (*b–d*) Images and per cent coverage of major benthic substrate categories observed at each site: algae, hard coral and other (e.g. sand, rock and soft coral).

The experiments were conducted at three shallow coral reef sites: Salt Pond (18.30806698° N, 64.70905197° W), Cocoloba (18.31528002° N, 64.76064996° W) and Tektite (18.309561998° N, 64.72218004° W) (figure 1). The sites were located at depths of 12.5 m (Tektite), 8 m (Cocoloba) and 5.1 m (Salt Pond). These sites were all relatively exposed on the southern shore of St John, with qualitatively similar hydrodynamic environments. The benthic composition, local fish communities and ambient soundscapes of these sites have been studied for several years [28,46–48]. Tektite has some of the highest levels of coral cover, fish density and species richness in the region, while Cocoloba and Salt Pond are both comparatively degraded sites with sparse coral cover and fewer fish. For the playback experiments, we considered Tektite as a ‘high quality’ reef and Cocoloba and Salt Pond to be ‘degraded’ reefs. Benthic substrates at the sites were visually surveyed and quantified prior to the experiment as part of a long-term monitoring study using 100-point 10 m SCUBA transects, following the methods outlined by Dinh *et al*. [49].

### 2.4. Acoustic playback system and treatments

**Figure 2.**
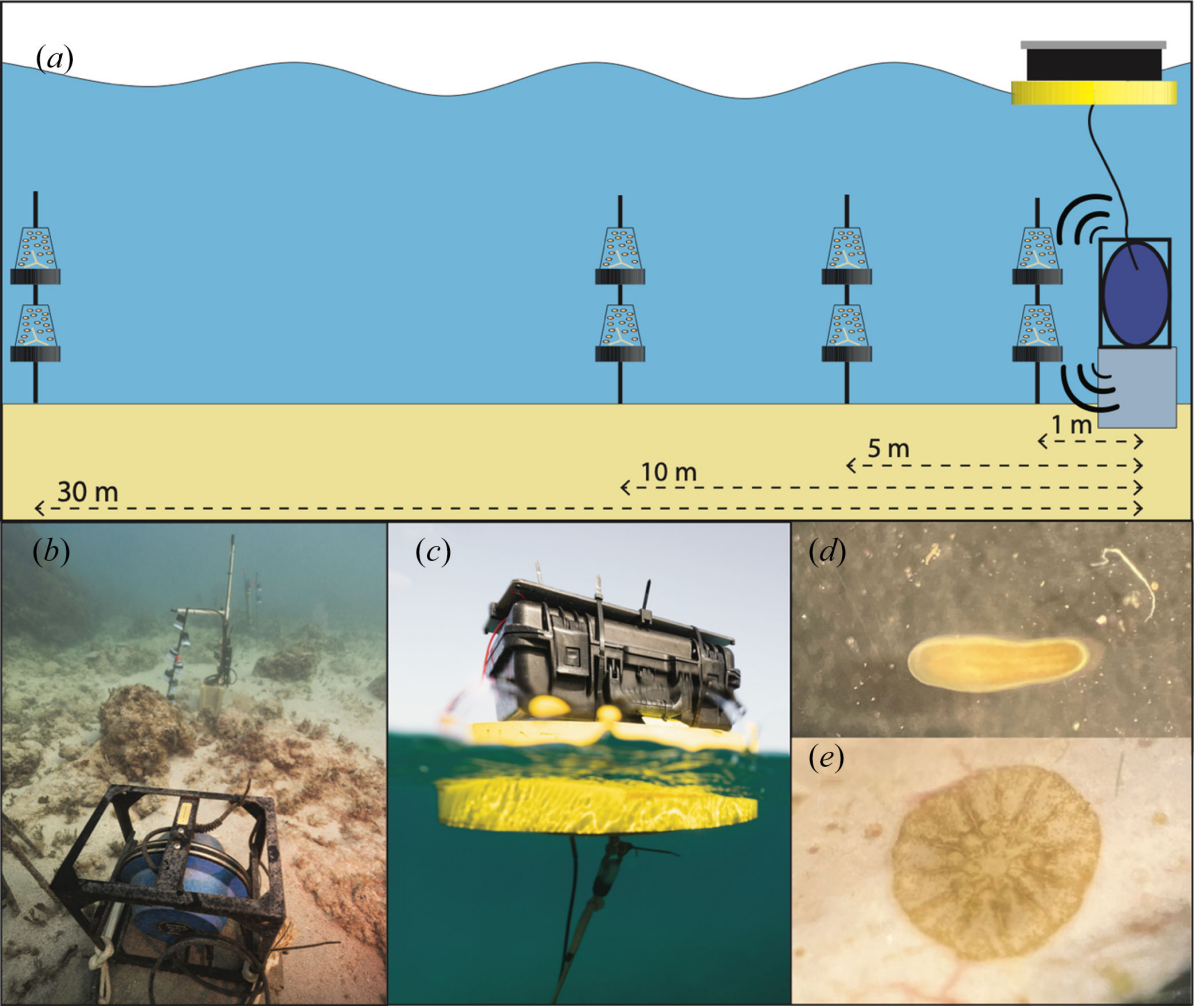
Overview of experimental set-up and study species. (*a*) Schematic of larval sound playback set-up. On the right, the RAPS is depicted with its benthic speaker mounted on a cinderblock and attached by a line to the surface buoy. Cups of larvae are illustrated and attached to stakes at progressive distances from the sound source. (*b*) RAPS speaker was photographed facing a rebar stake with attached cups of larvae and an orthogonal four-hydrophone recording array both placed 1 m from the speaker. (*c*) View of RAPS surface buoy including waterproof electronics housing and mounted solar panel. (*d*) *Porites astreoides* planktonic larva. (*e*) Settled *P. astreoides* larva on clay stilt.

We designed a custom acoustic playback system, the reef acoustic playback system (RAPS), to deliver underwater sound treatments. The RAPS consisted of an underwater speaker (Lubell Labs LL916C; frequency response 200 Hz–23 kHz; omnidirectional up to 2 kHz) fixed with cable ties to a cinderblock anchor on the seafloor and connected to a waterproof electronics housing mounted atop a surface buoy (figure 2*a–c*) [50]. The surface buoy was further secured by a rope connecting it to a secondary cinderblock mooring, which was placed on sand approximately 5 m from the speaker. The RAPS was powered from the surface by two 12 V LiFePO4 batteries, charged by a 17-watt 18-volt solar panel (Voltaic Systems) and managed by a solar charge controller (Victron Smartsolar). The timing of playback files was scheduled using a Teensy 4.0 microcontroller, connected sequentially to an AC transformer (Lubell Labs AC205C), a 220 watt amplifier (BOSS Audio Systems R1002) and the speaker (electronic supplementary material, figure S1).

Playback treatments of ‘healthy’ reef sounds consisted of four 12-h soundscape recordings taken from Tektite reef in August 2013, a time prior to the recent disturbances within the National Park of hurricanes Irma and Maria in 2017 and the outbreak of stony coral tissue loss disease (SCTLD) in 2020. A detailed description of these recording methods, as well as pre-hurricane characterization of the local soundscapes, can be found in the study by Kaplan *et al*. [28]. Each night of the experiment, one of the four 12-h soundscape recordings was randomly selected for playback. Files were synched to play at the same local time when they were recorded between 18.00 and 6.00. This 12-h overnight playback cycle was selected to allow the solar panels to recharge during the day and to ensure that the playback treatment would include crepuscular periods of fish chorusing.

Prior to both experiments, the propagation of the acoustic field produced by the RAPS was calibrated using a linear array of four 4-channel orthogonal hydrophones (HTI MIN-96, 2 Hz–30 kHz frequency response, sensitivities of −165.3 to −164.6 dB re 1 V/μPa), placed 1 m above the seafloor using a cinderblock base and connected to a SoundTrap (ST-4300, Ocean Instruments, 16-bit resolution) recorder sampling at a rate of 48 kHz. This allowed us to quantify both the pressure and particle motion components of the sound field [51]. Careful calibration of both components is critical, as sound pressure provides a commonly used acoustic variable of reference, but marine invertebrates are typically only sensitive to the particle motion component of sound [52].

### 2.5. Acoustic enrichment experiment

Playback experiments took place between 30 June–3 July and 30 July–2 August 2022. We enriched the soundscape at (i) the degraded reef site (Salt Pond) and compared rates of *P. astreoides* settlement within cups with those of (ii) the unenriched degraded reef (Cocoloba), where a RAPS system was placed and powered on but played no sound, and (iii) the higher-quality reef (Tektite), where no enrichment device was placed. The two unenriched sites served as prospective negative and positive controls for the effect of natural soundscapes on settlement. During the experiments, sound levels at each site were monitored at the four experimental distances from the speaker using either a four-channel hydrophone array (ST-4300; Ocean Instruments) or a single-channel recorder (ST-600; Ocean Instruments; hydrophone sensitivities of −176.4 to −175.9 dB re 1 V/µPa, 20 Hz–60 kHz frequency response, 16-bit resolution). Temperature and light levels at the three sites were monitored at 10 min intervals using pendant loggers (HOBO UA-002-64).

To determine the effect of sound enrichment, we compared settlement rates in three ways: between sites, with respect to time and as a function of distance from the speaker. Cups of larvae (*n* = 15 larvae per cup in June, *n* = 20 larvae per cup in July) were attached with cable ties to rebar stakes which had previously been hammered into the sand at distances of 1, 5, 10 and 30 m from (i) a speaker system playing reef sounds at the playback site, Salt Pond, (ii) a speaker powered on but not playing sound at Cocoloba or (iii) a marked central reef location at Tektite (we did not deploy a dummy system at Tektite so as to not confuse Park visitors with dive moorings in the same area). Thus, Salt Pond larvae received the replayed and enriched soundscape, Cocoloba received only the local degraded soundscape and Tektite received only the local, relatively ‘healthy’ natural soundscape. The larval cups, speakers and sound recorders were all placed in sand patches among or directly parallel to the existing reef structures to avoid damaging the benthos. Each month, two cups of larvae were placed at each of the four respective distances (*n* = 8 cups per site per month, *n* = 24 cups total per month). Cups were removed and examined for settlement after 72 h (figure 2).

In July, in order to investigate the fine-scale temporal impact of acoustic enrichment on larval settlement, an additional eight cups, each containing 10 larvae, were placed at Salt Pond and Cocoloba (four at each site) at 1 m from the speaker. Two cups per site were progressively removed from this distance at 24 and 48 h and scored for settlement. As noted above, all other cups from all three sites were scored after 72 h, providing a comparison of settlement after one, two or three nights of sound enrichment or control.

### 2.6. Data analysis

Cups and stilts were collected in the morning following three nights of acoustic enrichment and subsequently examined using a dissecting microscope (×40 magnification). Larvae were categorized as either swimming, settled or dead (figure 2*d,e*). Counts were excluded from analysis for any cups (10 cups total across both months) in which all added larvae could not be accounted for in one of these states. Differences in rates of larval settlement between sites and distances were compared using a one-way ANOVA followed by a Tukey multiple comparison of means tests using R Statistical Software v. 4.2.2 (R Core Team).

Acoustic recordings were analysed in MATLAB R2020a (The Mathworks Inc.), following the methods outlined by Jones *et al*. [51]. Mean power spectral densities (PSDs) were estimated (Hamming window, non-overlapping 0.5 s windows) from 1 min samples taken every 10 min across the total experimental length (62 h of recording including three nights (36 h) of acoustic playback). These PSDs were further subdivided into daytime and night-time recordings and plotted to compare the relative characteristics of the enriched daytime and unenriched night-time Salt Pond soundscape with the natural soundscapes of Tektite and Cocoloba. Particle accelerations across three axes of motion were calculated using a finite difference approximation method based on the difference in sound pressures between each pair of hydrophones in the four-hydrophone orthogonal arrays. Data were passed through a zero-phase digital filter with an eighth-order Butterworth filter and, subsequently, root-mean-square (RMS) levels were calculated for both sound pressure level (SPL_RMS_) and the three-dimensional vector norm of particle acceleration (PAL_RMS_) within a low-frequency band of 100–1000 Hz. This frequency band was chosen for analysis based on its overlap with the hearing ranges of reef fishes and invertebrates for which audiogram data exists [51]. PAL_RMS_ and SPL_RMS_ values were calculated and compared between four locations (1, 5, 10 and 30 m from the speaker) at Salt Pond and one location each at Tektite and Cocoloba.

Values of SPL_RMS_ and PAL_RMS_ recorded at 1, 5, 10 and 30 m from the speaker were used to estimate transmission losses (TL) of the acoustic signal produced by the RAPS. These data were averaged hourly over a 12-h playback period, and the averages for each hour were fitted to a nonlinear least squares regression model of the form: $a+blog_{10}r$ where *a* is the *y*-intercept, *r* is the distance (in m) from the sound source and the slope *b* represents the rate at which the intensity (in dB) of the sound source decreased owing to geometrical spreading [53]. In total, 24 empirical TL coefficients were estimated from SPL_RMS_ data and 24 were estimated from PAL_RMS_ data, and these values were compared with theoretical models of TL in underwater environments.

## 3. Results

### 3.1. Benthic environment and impacts of speaker playback on reef soundscape

**Figure 3.**
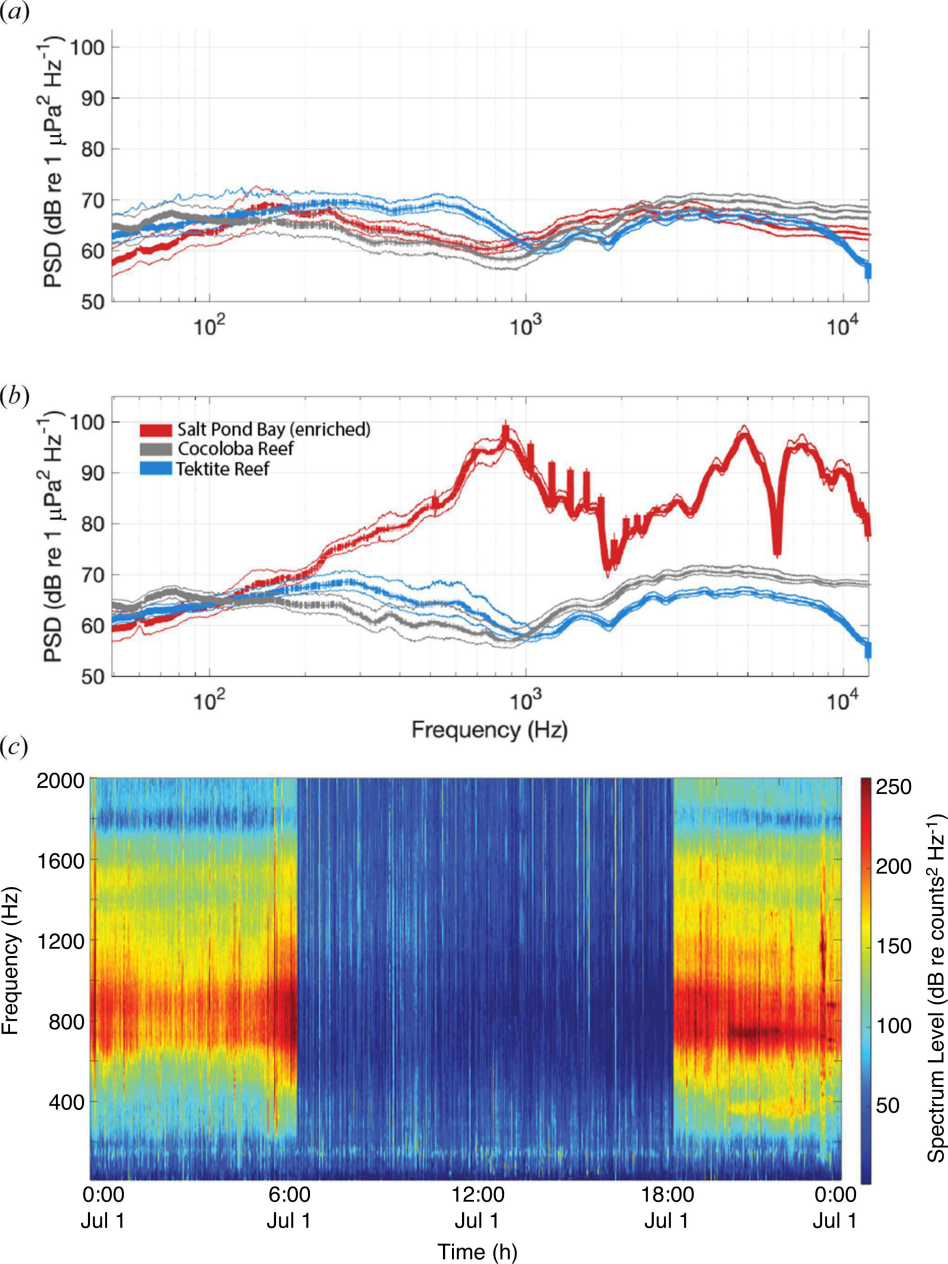
Natural and enriched soundscape features of the experimental sites. (*a*) PSDs of pressure values were recorded at the three reefs between 6.00 and 18.00 while no speaker was playing and only natural reef sounds were present (spectra calculated from recorders 1 m from source). (*b*) PSDs of pressure values were recorded at the three reefs between 18.00 and 6.00 while the speaker was playing only at Salt Pond (spectra calculated from recorders 1 m from source). (*c*) Long-term spectral average (LTSA) showing the low-frequency sound content of the Salt Pond soundscape 1 m from the speaker over a single 24-h period of the playback experiment (non-overlapping 5 s Hanning windows, nfft size = 4800). This LTSA was generated from uncalibrated pressure data using the Triton software package (Scripps Institution of Oceanography) for visualization purposes.

Benthic surveys indicated that the ‘high-quality’ site Tektite had over four times as much hard coral cover and less than one-quarter to one-third of the algal cover compared with the two ‘degraded’ sites (figure 1*b–d*). The unenriched soundscapes from all three sites showed expected similarities and differences in spectral characteristics based on these observed differences in reef health. Recordings from Tektite showed a peak in acoustic power between 300 and 800 Hz that was not seen at either Salt Pond or Cocoloba (figure 3*a*). The frequency composition of the Salt Pond and Cocoloba spectra were similar to each other, with the exception of the bands between 50–100 Hz and 4–12 kHz, where sound at Cocoloba was approximately 5 dB higher amplitude.

Playback from the RAPS speaker considerably altered both the shape and amplitude of the spectra at Salt Pond overnight within the 200–20 000 Hz frequency response range of the Lubell speaker, elevating the acoustic power below 1000 Hz by up to nearly 35 dB in the pressure domain compared with the unenriched recordings (figure 3*b,c*). The signature of the playback was audible and visible in spectrograms at all four playback distances between 1 and 30 m from the speaker.

### 3.2. Larval settlement results

Larvae settled at all sites and distances, primarily on the kiln stilts (0–60% larvae settled per cup), with occasional settlers observed on the cup lids (0–15%) and sides (0–25%; electronic supplementary material, table S1). Swimming larvae were observed in all chambers (20–93% larvae swimming per cup). Larval mortality was observed in 37% of the chambers and affected <20% of the larvae in all except one chamber, which saw the maximum observed value of 50% mortality.

**Figure 4.**
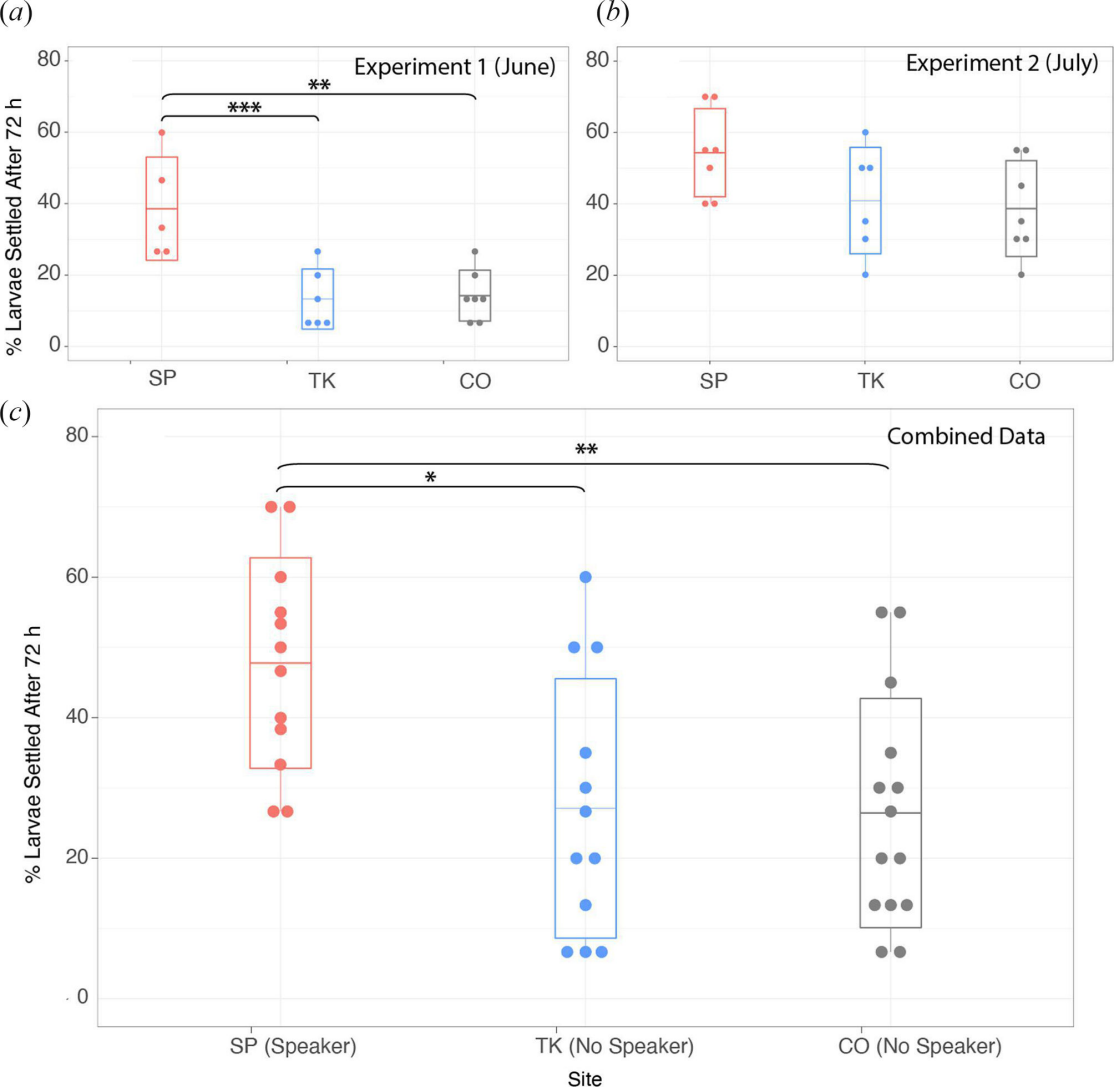
Results of acoustic enrichment experiment. Larval settlement rates per cup are shown from all distances and grouped by site for (*a*) June 2022 experiment, (*b*) July 2022 experiment and (*c*) combined data from both months. ANOVA significance codes: ***p < 0.001, **p <0.01, *p < 0.05.

The highest rates of settlement across both experimental months were measured at Salt Pond, the sound-enriched site (figure 4). In June, per-cup settlement rates at Salt Pond were significantly higher than both the degraded, non-enriched site, Cocoloba and Tektite, the healthiest site (ANOVA *F*_2,21_ = 15.24, *p* < 0.001), with an average of 39 ± 6% standard error (s.e.) settlement at Salt Pond, 13 ± 3% at Tektite and 14 ± 3% at Cocoloba (figure 4*a*). In July, settlement at Salt Pond remained higher than the unenriched sites, although the between-site differences during this month were not statistically significant (ANOVA *F*_2,29_ = 2.984, *p* = 0.0663; figure 4*b*). Notably, we collected more total larvae in July and all sites saw increased settlement compared to June, with Salt Pond averaging 54 ± 4%, Tektite 41 ± 6% and Cocoloba 39 ± 5%. Combining data from both months to address general trends, Salt Pond showed a significantly higher settlement rate than both Cocoloba and Tektite, with overall increases averaging 1.7 times more settlement under the sound enrichment treatment and maximum increases reaching seven times more settlement when comparing the lowest levels of observed settlement at the control sites (10%) with the highest settlement rates (70%) at Salt Pond (figure 4*c*).

### 3.3. Distance and sampling time effects on settlement rates

**Figure 5.**
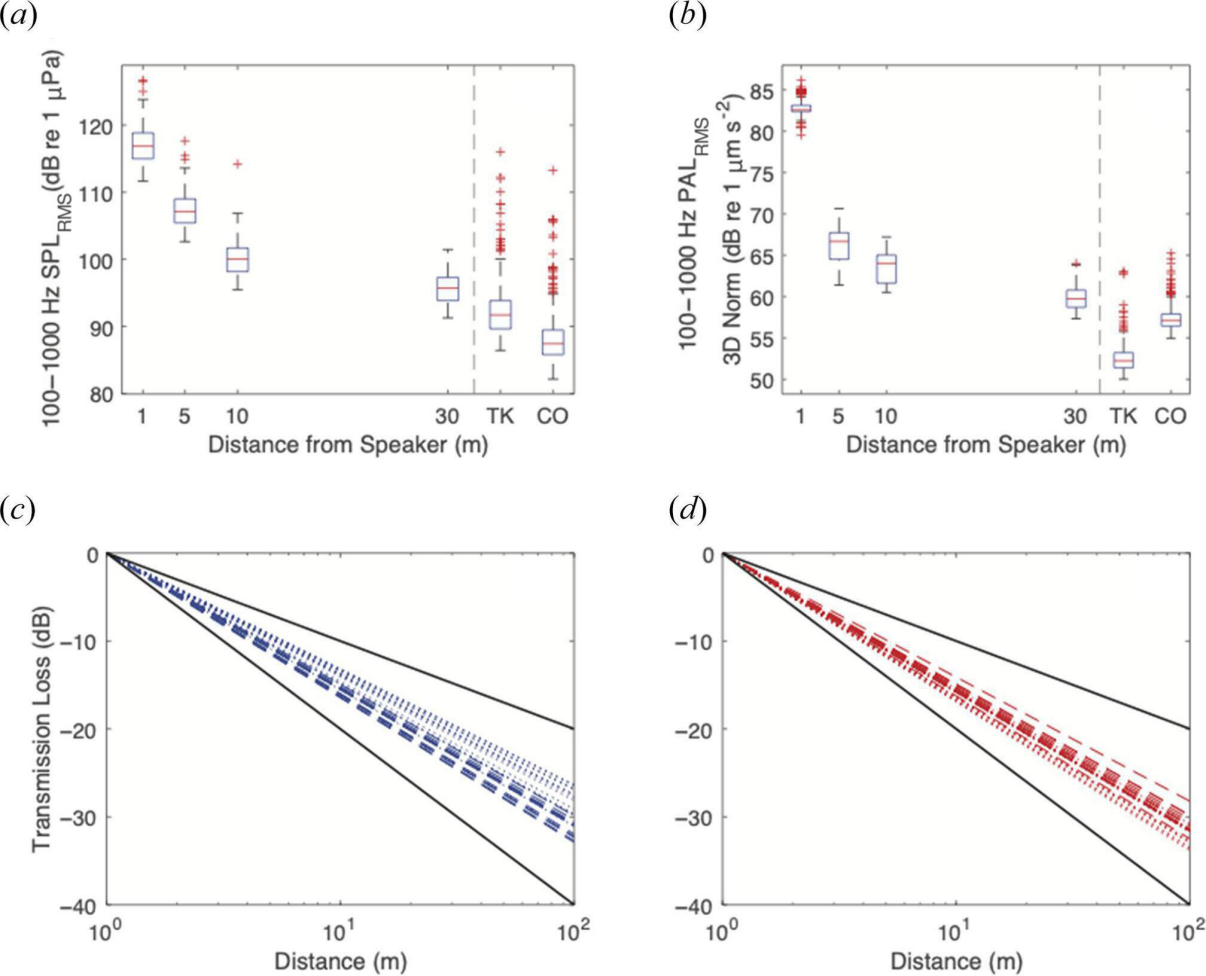
Propagation of the acoustic playback signal. (*a*) Boxplots of low-frequency RMS SPL were recorded at 1, 5, 10 and 30 m from the RAPS speaker at Salt Pond during a 12-h overnight acoustic playback period. Ambient values of SPL_RMS_ for Tektite and Cocoloba recorded throughout the July 3-day experimental period are shown to the right of the vertical dashed line. (*b*) Boxplots of PAL_RMS_ values calculated from the three-dimensional pressure data at Salt Pond, same times or locations as (*a*). (*c, d*) TL calculated from Salt Pond SPL_RMS_ (*c*) and PAL_RMS_ (*d*) values averaged hourly from two 12-h overnight calibration periods. Dashed lines are fitted to June’s calibration data, dotted lines are fitted to July’s data. Black lines represent two theoretical models for underwater TL: cylindrical (loss coefficient = 10) and spherical (loss coefficient = 20) spreading.

RMS levels of low-frequency SPL_RMS_ and PAL_RMS_ showed a rapid exponential decline with distance from the RAPS speaker, but at 30 m, acoustic levels remained higher than the median values recorded at Tektite and Cocoloba, where no speaker played sound (figure 5*a,b*). Empirically derived coefficients for TL ranged from −16.9 to −13.2, with all values falling intermediate between the theoretical coefficients of −10 for purely cylindrical spreading and −20 for spherical spreading (figure 5*c,d*).

**Figure 6.**
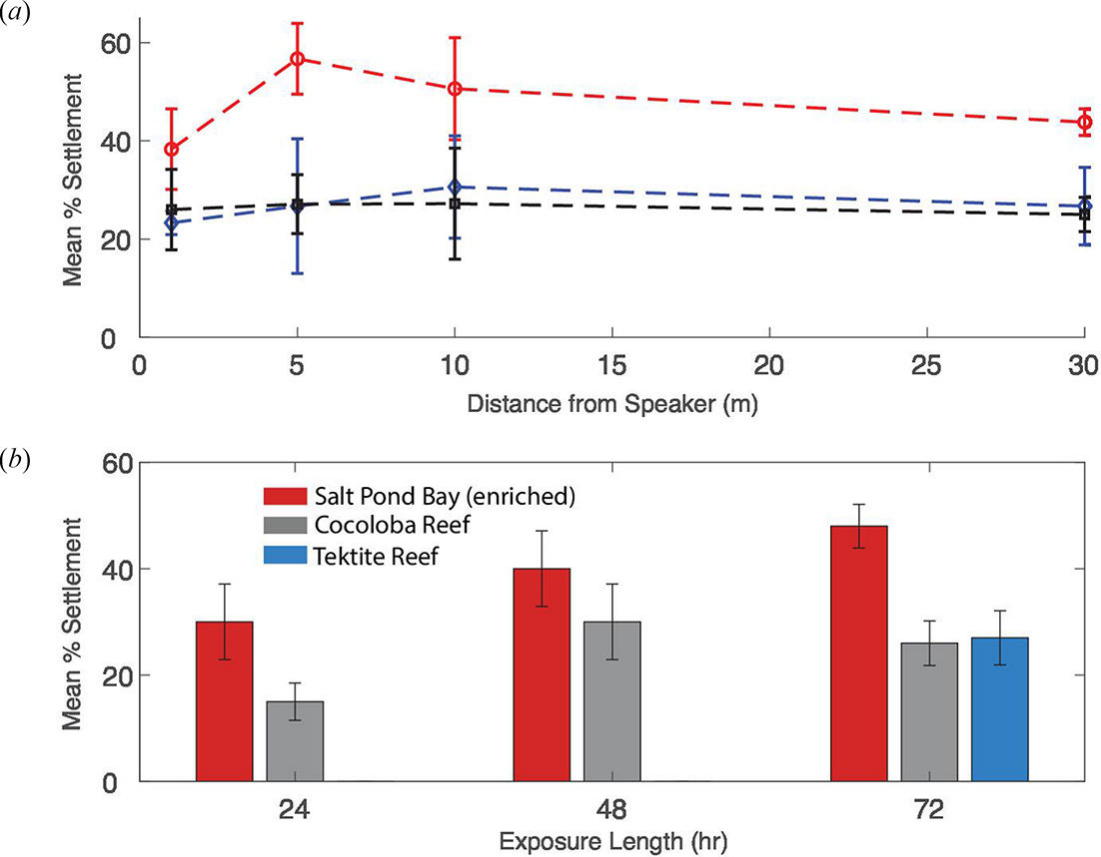
Larval settlement results grouped by distance and duration of exposure. Error bars = ±1 standard error. (*a*) Mean per-cup settlement at Salt Pond (red, circle), Tektite (blue, diamond) and Cocoloba (grey, square) graphed by distance from the speaker. Each data point corresponds to an average of between two and four cups of larvae. (*b*) Mean per-cup settlement at the three experimental sites graphed by time. Sample sizes for the 24- and 48-h time points were *n* = 2 cups of larvae per site. Sample sizes for the 72-h time point were *n =* 12 cups (Salt Pond), *n =* 14 (Cocoloba) and *n* = 12 (Tektite).

Larval settlement was higher at Salt Pond at all distances from the RAPS speaker compared with Tektite and Cocoloba (figure 6*a*). At Salt Pond, settlement was lowest (38 ± 8% s.e.) at 1 m, highest (57 ± 7% s.e.) at 5 m and showed a steady decrease between 5 and 30 m with increasing distance from the sound source. Tektite and Cocoloba showed lower but level settlement rates across all distances measured from the reef centre or dummy speaker, with average values ranging from 23 ± 2% s.e. to 31 ± 10% s.e.

Among the time-sampled treatment cups, mean settlement was two times higher at Salt Pond in cups sampled at 24 h and settlement remained elevated at Salt Pond compared with Cocoloba at both the 48- and 72-h time points. However, because the available larval supply restricted the sample size (*n* = 4) for the two early time points, these differences were not found to be significant using Student’s *t*‐test (*p* > 0.05; figure 6*b*).

## 4. Discussion

These results provide, to our knowledge, the first field-based study showing a positive effect of acoustic enrichment on coral larval settlement. We observed significantly higher rates of settlement of *P. astreoides* larvae at a degraded reef site with an enriched soundscape than on either high-quality or degraded natural reefs without an enriched soundscape. At the playback site, settlement rates depended on sound levels, which decreased with distance from the speaker, and the effect of sound enrichment on settlement was observed across multiple time points during the three-night exposure period.

While other studies have demonstrated variable settlement responses in coral larvae along naturally occurring gradients of sound [41,42], this is, to our knowledge, the first study to replicate these results using cues emitted by an introduced sound source. Because we observed both elevated rates of settlement at our playback site compared with our controls and decreasing levels of settlement with increasing distance from the sound source, we concluded that (i) our underwater speaker system was successful in delivering sound cues to *P. astreoides* larvae at ecologically relevant levels and (ii) our observed between-site differences in settlement were most likely owing to the presence of an enriched sound gradient.

This is also, to our knowledge, the first study to demonstrate increased settlement of invertebrate larvae placed at extended distances from a sound stimulus, up to 30 m away. Previous studies examining the effect of acoustic enrichment on oyster settlement in the field showed higher magnitudes of effect (up to 4–18 times more settlement with sound [37–39]). However, in these studies, all larvae were placed at a single distance less than 5 m from the sound source, and if replayed soundscapes were used, sound levels were 20–40 dB (10–100 times) higher than our levels at these close distances. Notably, in this study, we did not observe the highest settlement response in the chambers that were placed closest (1 m) to the speaker; settlement was, in fact, lowest at 1 m (though still elevated compared with control sites), highest at 5 m and showed a monotonic decrease at the further 10 and 30 m sites. This result could be an artefact of our limited sample sizes for each distance. It is also possible that the high amplitude of the sounds detected by the larvae closest to the speaker resulted in an inhibitory behavioural effect on settlement at the 1 m Salt Pond site. However, our lack of knowledge regarding the auditory sensory mechanism used by coral larvae and the optimal hearing ranges and detection thresholds for these organisms makes further conclusions on this point challenging.

We have provided thorough measurements of both received low-frequency SPL_RMS_ and PAL_RMS_ at all of our experimental playback distances (figure 5*a,b*). The goal is to provide baseline data for future studies to make a more precise determination of the thresholds and specific sound cues necessary to elicit behavioural responses in coral larvae. In shallow water environments with variable seabed surfaces, sound propagation often differs from theoretical estimates based on cylindrical or spherical models of spreading, as we demonstrated in our measurements of transmission loss at Salt Pond (figure 5*c,d*). Measuring settlement effects and PAL_RMS_ at multiple distances from a sound source should continue to be incorporated in acoustic enrichment studies, both to better parameterize the relationship between propagating sound stimuli and invertebrate behavioural responses, and to optimize the placement of acoustic playback devices on shallow reefs for maximum effects.

Somewhat surprisingly, we did not replicate previous observations of significantly higher settlement at our positive control site, Tektite, compared with our negative control site, Cocoloba, as found by Lillis *et al*. [42]. This result could potentially be explained by the recent ecological context of these sites. In the years since Lillis’ data were collected in 2017, Tektite Reef has faced numerous acute and chronic stressors including a major bleaching event and the previously mentioned SCTLD outbreak which has led to the rapid mortality of several massive coral species [54,55]. Our own surveys of Tektite have noted decreases in hard coral cover from 28.9% in 2017 to 19.8% in 2022 with concurrent increases in the presence of cyanobacterial mats and macroalgae (TA Mooney 2017–2022, unpublished data), including overgrowth of peyssonnelid algal crusts which threaten natural larval settlement [56]. Although we did not note major differences in the overall shapes of acoustic spectra recorded at Tektite and Cocoloba in 2022 compared with 2017 (figure 5*a*; electronic supplementary material, figure S2), Kaplan & Mooney [43] reported that hourly SPL_RMS_ levels in the low-frequency (100–1000 Hz) band at Tektite in 2013 ranged from 100 to 105 dB re 1 μPa. Our recordings showed a much lower median SPL_RMS_ value of 91.7 dB re 1 μPa in this same frequency range. Although the effect of these combined stressors on the composition of the natural acoustic settlement cues of Tektite has not been thoroughly investigated, these measurements suggest that low-frequency contributors to the reef soundscape, particularly fish sounds, have decreased appreciably over the past decade. Among our treatment sites, the settlement cups located 5 and 10 m from the speaker received SPL_RMS_ levels that most closely overlapped with 2013 Tektite sound levels, and these were also the cups which demonstrated the highest rates of settlement. This loss of significantly increased settlement in just 5 years between 2017 and 2022 underscores the severity of the threats these reefs face and the need for rapid, scalable solutions to enhance coral settlement and reef rebuilding.

Given the wide range of fertilization, larval duration and settlement strategies exhibited by corals, it is possible that acoustic cues may have variable effects depending on when in the competency period larvae are exposed to a cue. While settling early in the larval competency period may confer fitness advantages in larvae of other invertebrates [57], the effect of larval age at settlement on post-settlement process in corals is not well understood, though some species appear to be able to delay metamorphosis in the absence of settlement cues with no negative latent effects [58,59]. Here, we briefly investigated the timing component of the larval acoustic response by sampling a portion of cups from Salt Pond and Cocoloba at multiple time points throughout the 72-h evaluation period. Although our sample size for these early time points was small, the effect of the sound cue appeared evident within 24 h of exposure to sound playback and the magnitude of this effect persisted at the 48- and 72-h time points. It appears that *P. astreoides* larvae were responsive to the sound cue throughout the experiment, which is consistent with the life history of this species, whose competency period is known to last for days [60]. However, we did not collect enough data to sufficiently address additional questions, such as when in the larval developmental period did *P. astreoides* larvae begin responding to sound cues and whether there was a time point after which sound was no longer effective at inducing settlement above control levels. Future playback work would benefit by addressing these questions in additional coral species with varied larval characteristics, particularly vulnerable reef-building spawners such as acroporids and orbicellids. Such species-specific insights will allow restoration practitioners to better tailor the use of acoustic enrichment technology to the spawning characteristics of target species, ideally maximizing the ecological impact of this technology and minimizing the potential waste of committing resources to underwater speaker operation at suboptimal times and locations for settlement.

Taken together, our results suggest that the RAPS or similar playback systems could be a promising method to improve coral settlement success in combination with other restoration practises. We designed the RAPS system with the intent of providing a replicable tool that could be easily incorporated into existing systems of reef buoys and could serve a role in making restoration efforts visible (and audible) to tourists and reef stakeholders [50]. Avenues for specific implementations of this technology on coral reefs require careful consideration. We have shown that playback of reef sounds can significantly affect larval settlement in a situation where the available larval pool was controlled and isolated from other cue types and oceanographic forcings. In uncontrolled settings, the effect of sound may be less pronounced. For example, in reef fishes, acoustic enrichment can increase the settlement of certain species, but ocean currents can play a strong, sometimes dominating, role in spatially redistributing the larvae and affecting the small-scale process of settlement [49]. Therefore, other factors, such as the hydrodynamic variability and the associated inhomogeneity in larval dispersal, need to be taken into consideration when applying acoustic enrichment *in situ*.

Furthermore, while consensus is emerging that sound is an important settlement cue for marine fishes and invertebrates, these animals clearly sense and respond to multiple cues, which may result in synergistic, antagonistic or additive interactions. Acoustic playback should be integrated and evaluated alongside other cue types such as chemicals and light [11] to address how using multiple sensory modalities may further enhance settlement. Such research could yield insights as to whether altering the soundscape can compensate for a lack of cues in other sensory modes, or vice versa, with implications for restoration practise. Acoustic enrichment may not be a substitute for chemical cues but could be used in combination with chemical conditioning to accelerate, scale and improve the efficiency of existing nursery or out-planting practises. A benefit of this system is that the sound cue can play continuously (or in a scheduled nature), not dissipating over time or with hydrodynamic influences.

As with reef restoration efforts more generally, there are potential drawbacks to inducing settlement at degraded sites. Shepherding coral larvae to long-term success at restoration sites involves significant effort and periodic monitoring of sites for months to years [61]. If conditions are not optimal for juvenile growth and survival, even if settlement is successful, larvae may be quickly overgrown by algae, covered by sediment, predated upon or for other reasons fail to survive long enough to become reproductive or restore key community functions [62]. Yet under circumstances where there is a paucity of natural cues, encouraging settlement could still give these animals a potential ‘head start’ to growth and later life stages, especially when the likely alternative outcomes are either larval mortality in the water column or ‘desperate’ settlement in suboptimal conditions after larvae have exhausted their lipid resources [63].

The significant costs associated with even small-scale reef restoration, and implementation of acoustic enrichment technology will require thoughtful consideration of the optimal locations for deployment and how best to limit post-settlement mortality. Since reef sound cues are unlikely to propagate far enough to attract larvae from extended distances >1 km [43], RAPS will only be effective in regions where there are sufficient pools of nearby larvae. These larvae could be produced by local populations of corals, delivered by ocean currents from spawning grounds some distance away, or actively seeded for restoration purposes [64,65]. Future acoustic enhancement initiatives can maximize their value by incorporating knowledge of reef community structure and local hydrodynamics to target areas for enrichment where larvae may already be being naturally transported but settlement and recruitment are not successful owing to deficiencies in the local cuescape.

Finally, as previously noted by McAfee *et al*. [39], studies of acoustic enrichment to date have broadly been confined to assessing effects at the single species, taxon group or trophic level. Since many reef fishes and invertebrates are now known to respond to sound, future studies would benefit from quantifying the effects of acoustic enrichment at the community level using added techniques such as video observation. Acoustic enrichment has the potential to lead to positive feedback effects on reefs over broad spatial and temporal scales, if the larval fishes and invertebrates that respond to playback subsequently produce additional cues to continue attracting further members to the community [35]. This could, in turn, lead to increases in community interactions such as predation, which the larvae in our study were protected from but realistically is a significant risk factor affecting the survival of pre-settlement corals. Understanding the role of sound in the context of other settlement cues, hydrodynamic conditions and community interactions present on coral reefs is a rich area of study for the future. Overall, since corals provide the physical framework for tropical reef ecosystems, this demonstration of acoustically enriched coral settlement suggests a key means to support these critical species and assist in rebuilding reef habitats.

## 5. Conclusion

We demonstrated in a field setting that acoustic enrichment of a degraded coral reef can induce and increase settlement of *P. astreoides*, coral larvae. Settlement rates remained elevated at ecological and restoration-relevant distances, generally weakening with increasing distance from the acoustic source, though our results suggest that settlement may continue to be elevated at distances greater than our maximum measured radius of 30 m. Furthermore, at least with *P. astreoides*, a brooding species, the settlement was also higher throughout the critical first 72 h of competency when larvae were exposed to a sound source. The observation that settlement may be mediated by manipulating local soundscapes is a new insight into coral larval behaviour, providing a malleable means to address settlement ecology *in situ*. Coral reefs are urgently in need of innovative solutions to rapidly restore lost ecosystem functions and cues. These initial results suggest acoustic enrichment supports vital settlement activities on coral reefs and can be an effective tool to speed reef recovery.

## Data Availability

The datasets supporting this article have been uploaded as part of the electronic supplementary material [66].
